# Palmitoylation of SARS-CoV-2 S protein is essential for viral infectivity

**DOI:** 10.1038/s41392-021-00651-y

**Published:** 2021-06-11

**Authors:** Zhuanchang Wu, Zhaoying Zhang, Xin Wang, Jing Zhang, Caiyue Ren, Yuming Li, Lifen Gao, Xiaohong Liang, Peihui Wang, Chunhong Ma

**Affiliations:** 1 Key Laboratory for Experimental Teratology of Ministry of Education and Dept. Immunology, School of Basic Medical Sciences, Cheeloo Medical College, Shandong University, Jinan, Shandong China; 2 College of Agriculture and Forestry, Linyi University, Linyi, Shandong China; 3 Advanced Medical Research Institute, Shandong University, Jinan, Shandong China; 4 State Key Laboratory of Respiratory Disease, National Clinical Research Center for Respiratory Disease, Guangzhou Institute of Respiratory Health, The First Affiliated Hospital of Guangzhou Medical University, Guangzhou, China; 5 Key Laboratory of Infection and Immunity of Shandong Province, Shandong University, Jinan, Shandong China; 6 Collaborative Innovation Center of Technology and Equipment for Biological Diagnosis and Therapy in Universities of Shandong, Jinan, Shandong China

**Keywords:** Microbiology, Pathogenesis

**Dear Editor**,

Severe acute respiratory syndrome coronavirus 2 (SARS-CoV-2) is the causative agent of the unprecedented coronavirus disease 2019 (COVID-19). SARS-CoV-2 entry into host cells is mediated by the viral transmembrane spike (S) glycoprotein that forms homotrimers protruding from the viral surface. S protein comprises two functional subunits S1 and S2, responsible for binding to host receptor angiotensin-converting enzyme 2 (ACE2) and the membrane fusion of SARS-CoV-2 with host cell membranes, respectively. Membrane fusion is necessary for release of viral genome RNA into the host cell cytoplasm. The entry of SARS-CoV-2 is a complex process that requires the concerted action of receptor-binding and proteolytic processing of S protein to promote virus-cell fusion.^1^ After cleavage, the heptad repeat 1 (HR1) and 2 (HR2) domains in S2 subunit interact with each other to form a six-helix bundle (6-HB) fusion core and insert into the target cell membrane, thus bringing the viral and cell membrane into close apposition for fusion and infection.^2^ However, regulatory mechanisms of S-mediated membrane fusion are still less known.

Protein palmitoylation is a common covalent fatty acid modification that occurs on cytoplasmic cysteine residues with a 16-carbon fatty acid palmitate, catalysed by a family of zinc finger DHHC domain-containing protein palmitoyltransferases (ZDHHCs), of which 24 members (ZDHHC1–24) have been identified in mammals. Palmitoylation enhances protein hydrophobicity and plays important roles in the regulation of protein subcellular localization, trafficking, stability and interaction with other proteins. Palmitoylation of viral proteins is known to be involved in virus assembly and infection. The S protein of SARS-CoV-1 has been shown to be palmitoylated that appear to be important for cell–cell fusion,^3^ whether S palmitoylation is critical for SARS-CoV-2 infection and its regulatory mechanism are still elusive.

To explore the palmitoylation of SARS-CoV-2 S protein, we performed an acyl-biotin exchange (ABE) assay.^4^ As shown in Fig. 1a, ectopically expressed S protein was clearly acylated. The ABE assay efficiency was verified by detection of NOD2 palmitoylation as reported^4^ (Supplementary Fig. S1a). ZDHHC5 and GOLGA7, the acyl-transferase complex mediates protein palmitoylation, are reported to be physically associated with SARS-CoV-2 S protein.^5^ To confirm the interactions of S protein with ZDHHC5-GOLGA7, Flag-S, HA-ZDHHC5 and Myc-GOLGA7 were coexpressed in HEK293T cells. Co-immunoprecipitation (Co-IP) assays demonstrated that S protein interacts with both ZHHHC5 and GOLGA7, vice versa (Fig. 1b). Further ABE assay found that ectopic ZDHHC5 obviously increased the palmitoylation level of S protein, which was further enhanced by overexpression of GOLGA7 (Fig. 1c). By contrast, a catalytically inactive ZDHHC5 mutant (ZDHHC5-C134S) abolished ZDHHC5-induced upregulation of S palmitoylation (Fig. 1d). These data indicate that S protein is palmitoylated by the ZDHHC5-GOLGA7 complex.

**Fig. 1:**
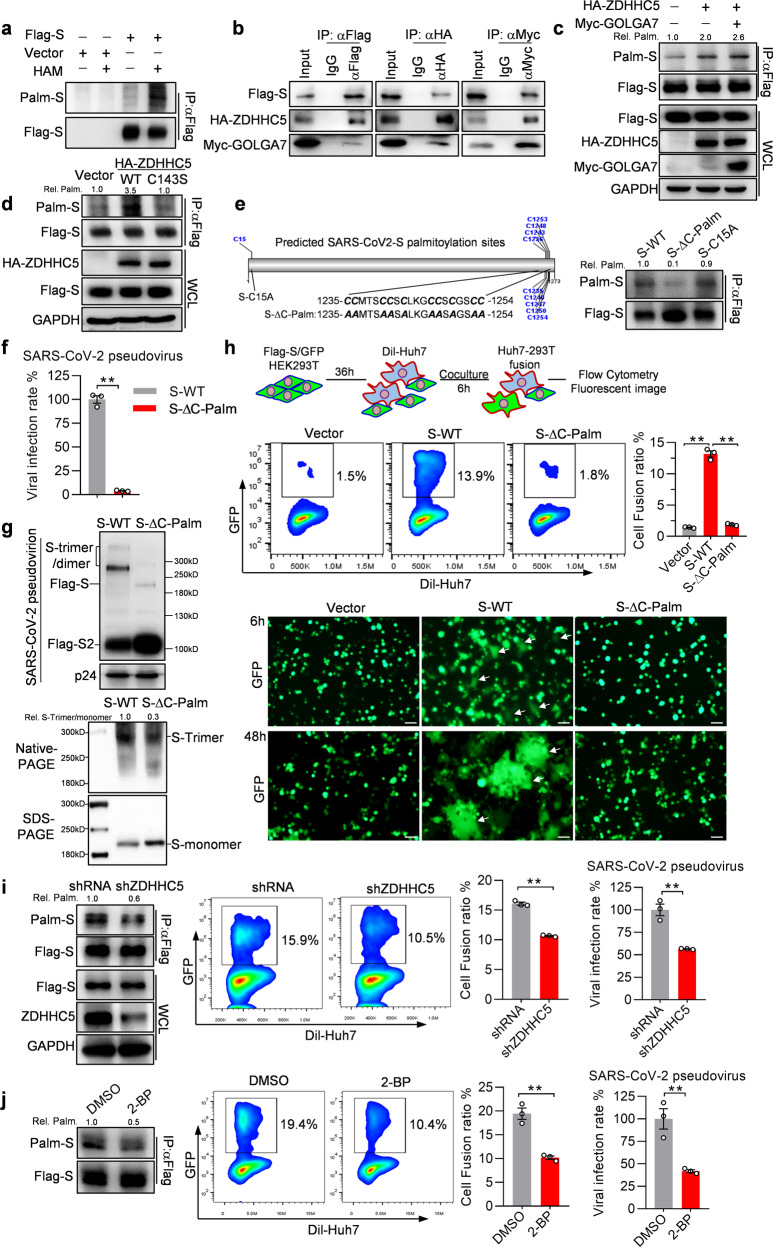
Palmitoylation of SARS-CoV-2 S protein contributes to membrane fusion and viral infection. **a** SARS-CoV-2 S protein is palmitoylated. Flag-S was transfected into HEK293T cells, 48 h later, its palmitoylation was detected by ABE assay in the presence of hydroxylamine (HAM). **b** ZDHHC5 and GOLGA7 interact with S protein. Flag-S, HA-ZDHHC5 and Myc-GOLGA7 constructs were cotransfected into HEK293T cells, 48 h later, protein interactions were measured by Co-IP. **c** ZDHHC5 and GOLGA7 contribute to S protein palmitoylation. Flag-S was coexpressed with HA-ZDHHC5 or HA-ZDHHC5/Myc-GOLGA7 in HEK293T cells for 48 h, then the palmitoylation levels of S were detected. **d** The palmitoyltransferases activity of ZDHHC5 is essential for regulating S protein palmitoylation. Flag-S plasmid was cotransfected with HA-ZDHHC5 or HA-ZDHHC5-C143S plasmids into HEK293T cells, 48 h later, the palmitoylation levels of S were measured by ABE assay. **e** The palmitoylation sites of S protein are Cys residues at C-terminus. The palmitoylation sites of S were predicted by CSS-Palm tool. The blue fonts indicated the predicated palmitoylation sites (left panel), and the palmitoylation levels of wild-type S, C-C15A and S-∆C-Palm mutants were measured by ABE assay (right panel). **f** Palmitoylation of S protein is required for the infectivity of SARS-CoV-2 pseudoviruses. HEK293T-ACE2 cells were infected with lentiviruses pseudotyped with S-WT or S-∆C-Palm for 72 h, viral infection rate was analysed through detecting firefly luciferase activity relative to the level (set as 100) at S-WT (*n* = 3). Unpaired *t*-test, *P < 0.05; **P < 0.01. **g** S-trimer formation depends on its palmitoylation. Lentiviruses pseudotyped with S-WT or S-∆C-Palm were packaged from HEK293T cells and purified through supercentrifuging under 20% sucrose cushion, then S protein expression on pseudoviruses particles was detected by western blot, HIV-1 p24 antigen as loading control (upper panel). S-WT and S-∆C-Palm were overexpressed in HEK293T for 48 h, the relative S-trimer/monomer levels were detected by western blot (lower panel). **h** Palmitoylation of S protein is essential for S-mediated cell–cell fusion. S/GFP and S-∆C-Palm/GFP coexpressed HEK293T cells were cocultured with Dil-labelled Huh7 cells, cell fusion was measured with flow cytometry (*n* = 3) and visualized by fluorescent imaging at indicated time. The scale bar indicates 50 µm. One-way ANOVA, *P < 0.05; **P < 0.01. **i** ZDHHC5 knockdown inhibits SARS-CoV-2 pseudovirus infection. HEK293T cells were transfected with shZDHHC5 for 24 h, Flag-S plasmid alone or together with other packing plasmids were transfected into these cells. Another 48 h later, the SARS-CoV-2 pseudoviruses were collected to infect HEK293T-ACE2 cells. S palmitoylation levels were measured by ABE assay. S-mediated cell–cell fusion and SARS-CoV-2 pseudoviruses infection rate of HEK293T-ACE2 cells were detected as in 1h and 1f (*n* = 3). Unpaired *t*-test, *P < 0.05; **P < 0.01. **j** 2-BP represses SARS-CoV-2 pseudoviruses infection. HEK293T cells were transfected with Flag-S alone or together with other packing plasmids for 12 h and subsequently treated with 2-BP at 25 μM for another 36 h. S protein palmitoylation levels were measured by ABE assay. S-mediated cell fusion and pseudovirus infection rate were detected as in 1h and 1f (*n* = 3). Unpaired *t*-test, *P < 0.05; **P < 0.01

We employed the motif-based palmitoylation sites predicator CSS-palm 4.0 (http://csspalm.biocuckoo.org/online.php) to identify the palmitoylation site of S protein. A single palmitoylation site (C15) at N terminus and nine Cys sites within cytosolic C-terminus domain of S protein were predicted (Fig. 1e, left panel). Compared to wild-type S protein (S-WT), substitution of Cys residues at C-terminus by alanine (S-∆C-Palm) completely abolished the palmitoylation of S protein, while mutant with Cys to Ala at C15 (S-C15A) cannot affect the palmitoylation of S protein (Fig. 1e, right panel).

To further investigate the role of S palmitoylation in SARS-CoV-2 infection, luciferase-expressing pseudoviruses bearing S-WT or S-∆C-Palm were generated in the envelope-defective HIV-1 backbone (Supplementary Fig. S1b). The pseudovirus entry efficiencies were estimated by analysing the levels of luciferase activities in human ACE2-expressing HEK293T cells (HEK293T-ACE2). Compared to S-WT pseudovirus, S-∆C-Palm pseudovirus showed an approximate 25-fold decrease of luciferase activities in HEK293T-ACE2 cells (Fig. 1f), suggesting that the entry of SARS-CoV-2 pseudovirus is highly dependent on S protein palmitoylation. To investigate whether palmitoylation affects the abundance of S protein in the SARS-CoV-2 pseudovirions, we purified pseudovirions by sucrose density gradient ultracentrifugation. As shown in Fig. 1G upper panel, SARS-CoV-2 S protein had been cleaved during viral packaging and ∆C-Palm did not decrease the abundance of total S protein packaged into the pseudovirions. Interestingly, we detected more cleaved S2 subunits and less dimeric/trimeric S proteins (>250 kD) in the pseudovirions with S-∆C-Palm than that of pseudovirions with S-WT. In accordance, with native PAGE, significantly reduced trimer formation of S protein was also detected in S-∆C-Palm mutant expressing cells than S-WT expressing cells (Fig. 1g, lower panel). Palmitoylation is known to regulate protein trafficking and palmitoylation of SARS-CoV-1 S protein is reported to promote its distribution in the detergent-resistant membranes.^3^ However, compared to S-WT, we did not observe the reduction of S-∆C-Palm partitioning into cell membrane and lipid raft fractionations (Supplementary Fig. S1c), excluding the possible involvement of palmitoylation in the membrane trafficking of S protein.

The fusion between viral and cellular membrane is a critical step for viral entry; thus, we further explored whether palmitoylation of S protein determines the membrane fusogenic capacities of SARS-CoV-2 pseudovirus. S-mediated cell–cell fusion assays were performed by incubating Flag-S and GFP co-expressing HEK293T cells with Dil-labelled Huh7 target cells. Flow cytometric analysis showed that after 6 h incubation, about 18% of Huh7 cells fused with S-WT/GFP coexpressed HEK293T cells displaying as GFP positive signals in Dil-labelled Huh7 cells. This cell–cell fusion was almost completely destroyed in Huh7 cell incubating with S-∆C-Palm/GFP co-expressing HEK293T cells (Fig. 1h). Similar results were obtained with fluorescent imaging in coculture of S/GFP co-expressing HEK293T cells and Huh7 cells. At 6 h post coculture, cell–cell fusion verified as larger morphology and weaker fluorescence intensity of GFP (white arrows) was observed in S-WT/GFP co-expressing HEK293T cells but not in empty vector control and S-∆C-Palm/GFP co-expressing HEK293T cells (Fig. 1h). Even 48 h later, there were still no fused cells in S-∆C-Palm and empty vector cocultured groups, while big syncytia appeared in wild-type S expressing HEK293T cells (Fig. 1h). Consistently, with Dil-labelled HEK293T-ACE2 as target cells, flow cytometry and fluorescent imaging confirmed the deficiency of cell–cell fusion and syncytia formation in S-∆C-Palm mutant expressing cell–cell cocultures (Supplementary Fig. S1d). Collectively, these data suggest that S palmitoylation is essential for SARS-CoV-2 infectivity by controlling S protein trimer formation and subsequently membrane fusion.

Since depalmitoylation displayed significant inhibition of SARS-CoV-2 pseudovirus entry, we further asked whether targeting palmitoylation of S protein is a potential therapeutic strategy. To this end, we reduced ZDHHC5 expression by shRNA targeted knockdown. As predicted, ZDHHC5 knockdown deceased the palmitoylation of S protein (Fig. 1i). Moreover, ZDHHC5 knockdown caused significant defects in S-mediated cell–cell fusion and pseudovirus entry (Fig. 1i). In accordance, treatment of palmitate analog 2-brompalmitate (2-BP) (25 μM), a general protein palmitoylation inhibitor, led to about half decrease of palmitoylation levels of S protein (Fig. 1j) without any cytotoxicity in HEK293T cells (Supplementary Fig. S1e). Also, palmitoylation levels of S protein were reduced by 2-BP treatment (Fig. 1j), which simultaneously repressed S-medicated membrane fusion rate of HEK293T-Huh7 cells and infectivity of SARS-CoV-2 pseudovirus (Fig. 1j). Together, these results suggest that intervening palmitoylation of S protein contributes to restricting SARS-CoV-2 transmission.

Our study reveals the critical role of palmitoylation of SARS-CoV-2 S protein in controlling membrane fusion and virion infectivity. ZDHHC5 and GOLGA7 are found to enhance SARS-CoV-2 S protein palmitoylation synergistically. More importantly, targeting palmitoylation of S protein by knockdown ZDHHC5 or treatment with a protein palmitoylation inhibitor 2-BP suppresses S-mediated membrane fusion and the entry of SARS-CoV-2 pseudovirus into host cells. Consistent with our findings, two latest reports also found that Cys sites within cytosolic C-terminus domain of S protein are required for cell–cell fusion and palmitoylated by ZDHHC5,^6,7^ which together with our work strongly suggest a novel insight into blocking S palmitoylation for controlling SARS-CoV-2 infection. It is worth noting that S-∆C-Palm mutation led to no changes in the membrane and lipid raft distribution of SARS-CoV-2 S protein, but this mutation resulted in obvious decrease of S-trimer formation, which may explain the critical role of palmitoylation in controlling S-mediated membrane fusion and SARS-CoV-2 pseudovirus infection. However, how palmitoylation affects S protein trimerization is not clear, which needs to be further investigated in future studies.

## Supplementary information

Clean Supplementary Material

## Data Availability

Further information and requests for resources and reagents should be directed to and will be fulfilled by the Lead Contact, Chunhong Ma (machunhong@sdu.edu.cn) and Peihui Wang (pei-hui.wang@connect.hku.hk).
