# Supplementary material for: Palmitoylation of SARS-CoV-2 S protein is essential for viral infectivity
Source: Signal Transduct Target Ther. 2021 Jun 11;6:231. doi: 10.1038/s41392-021-00651-y (PMC8193602; doi:10.1038/s41392-021-00651-y)
Supplement: Supplementary file 1 — Clean Supplementary Material [file 41392_2021_651_MOESM1_ESM.docx]

**Supplemental Material for**

**Palmitoylation of SARS-CoV-2 S Protein Is Essential for Viral Infectivity**

Zhuanchang Wu^1,†, *^, Zhaoying Zhang^1,†^, Xin Wang^5,†^, Jing Zhang^2,†^, Caiyue Ren^1^, Yuming Li^6^, Lifen Gao^1,3,4^, Xiaohong Liang^1,3,4^, Pei-Hui Wang^2,*^ and Chunhong Ma^1,2,3,4,*^

^1^Key Laboratory for Experimental Teratology of Ministry of Education and Dept. Immunology, School of Basic Medical Sciences, Cheeloo Medical College, Shandong University, 250012 Jinan, Shandong, China; ^2^Advanced Medical Research Institute, Shandong University; ^3^Key Laboratory of Infection and Immunity of Shandong Province, Shandong University, 250012 Jinan, Shandong, China; ^4^Collaborative Innovation Center of Technology and Equipment for Biological Diagnosis and Therapy in Universities of Shandong; ^5^College of Agriculture and Forestry, Linyi University, Linyi, Shandong, China; ^6^State Key Laboratory of Respiratory Disease, National Clinical Research Center for Respiratory Disease, Guangzhou Institute of Respiratory Health, the First Affiliated Hospital of Guangzhou Medical University, 510182 Guangzhou, China.

^†^ These authors contributed equally to this work

^*^ Correspondence: Chunhong Ma ([machunhong@sdu.edu.cn](mailto:machunhong@sdu.edu.cn)), Zhuanchang Wu ([wujichang19861213@163.com](mailto:wujichang19861213@163.com)) and Pei-Hui Wang ([pei-hui.wang@connect.hku.hk](mailto:pei-hui.wang@connect.hku.hk))

**Supplementary information and Figures**

Supplementary materials and methods.

Fig. S1 The cell-cell fusion mediated by S protein.

**Materials and methods**

**Method Details**

***Cells and Plasmids***

The human embryonic kidney cell line 293T (HEK293T) and the human hepatoma cell line Huh7 were purchased from the Shanghai Cell Collection (Shanghai Institutes for Biological Sciences, Chinese Academy of Sciences, Shanghai, China). HEK293T-ACE2 cell line was established to stably express human ACE2 receptor. All cell lines were cultured in Dulbecco’s Modified Eagle’s Medium (DMEM) with 10 % fetal bovine serum (FBS). Transfections of HEK293T were performed using Lipofectamine 2000 (Thermo Fisher Scientific, USA) according to the manufacturer’s protocol.

The SARS-CoV-2 S protein gene sequence (NCBI accession no. MN908947) was synthesized (General Biol, China) and subcloned into the pCAG-Flag expression vector (Flag-S). Plasmids expressing HA-tagged human ZDHHC5 (HA-ZDHHC5) and Myc-tagged human GOLGA7 (Myc-GOLGA7) were generated by inserting full-length human ZDHHC5 and GOLGA7 coding sequence into the pcDNA3.0 vector, respectively. S-C15A, S-∆C-Palm and ZDHHC5-C143S site mutants were generated by KOD-Plus-Mutagenesis Kit (TOYOBO, Japan). Flag-NOD2 plasmid, shZDHHC5 plasmid (5’-CCCACATTATGGGTGTGTT-3’) and shRNA plasmid (5’- TTCTCCGAACGTGTCACGT-3’) were friendly gifted by Dante Neculai (Zhejiang University School of Medicine).

***Cell-cell fusion assays***

Flag-S and pEGFP-N1 plasmids at 2:1 ratio were cotransfected into HEK293T effector cells. Huh7 cells naturally expressing ACE2 receptors and HEK293T-ACE2 cells labeled with Dil dye (Thermo Fisher Scientific) at 2 μM were used as target cells. 4 × 10^4^ target cells (Huh7 and HEK293T-ACE2) were incubated in 24-well plates at 37 ℃ for 4 h, followed by the addition of 20 × 10^4^ HEK293T effector cells. After coculture for 6 h, fused cells were analyzed by flow cytometry or observed under an inverted fluorescence microscope. After coculture for 24 h and 48 h, syncytium formation was observed under the inverted fluorescence microscope.

***Acyl-Biotin Exchange (ABE) assay***

The ABE assay was performed as previously described^1^. In brief, HEK293T cells transiently expressing Flag-S were harvested 48 h after transfection and washed with cold PBS. For the ZDHHC5 knockdown experiments, the shZDHHC5 plasmid was transfected 24h before the transfections of the Flag-S plasmids. Before cell lysis, Nethylmaleimide (NEM) was dissolved in 100% EtOH and added to the lysis buffer (50 mM Tris-HCl pH 7.5, 150 mM NaCl, 1 mM MgCl_2_, 1% NP-40, 10% glycerol) with protease inhibitor to a final concentration of 50 mM. Cells were then suspended in NEM containing lysis buffer for 1 h on ice and the supernatants were incubated with anti-flag beads (Bimake, B23102) at 4 ℃ for 4 h. After incubation, the beads were washed four times with Lysis Buffer-pH 7.5 and then three times with Lysis Buffer-pH 7.2. Then, beads were incubated with a freshly prepared hydroxylamine (HAM)-containing lysis buffer (pH 7.2, 1M HAM) with protease inhibitor at room temperature for 1 h and washed four times with Lysis Buffer-pH 7.2 and three times with Lysis Buffer-pH 6.2. Subsequently, beads were treated with Biotin-BMCC-containing Lysis Buffer (pH 6.2, 5μM Biotin-BMCC) at 4 ℃ for 1 h. The immunoprecipitate samples were analyzed by western blot using anti-Flag antibody (1:5000; MBL, Japan) and streptavidin-HRP (1:5000, Beyotime, China).

***Production and infection of SARS-CoV-2 S pseudoviruses***

To generate the SARS CoV-2 S pseudotyped HIV-1 single-round luciferase virus, 4 × 10^6^ HEK293T cells were co-transfected with pLP1, pLP2, pCDH-CMV-Luciferase-CopGFP and pCAG-SARS-CoV-2-S∆C19 using the Lipofectamine 2000 transfection reagent according to the manufacturer’s instructions. The cells were transferred to fresh DMEM 12 h later. The supernatant containing SARS-CoV-2 pseudoviruses were harvested 48 h after transfection, and then centrifuge the viral supernatant at 5000 g for 3 min to pellet any cell debris and filtered through a 0.45 μm filter. 200 μl viral supernatant was used to extract viral RNA and digested with DNase I to eliminate plasmid contamination, and viral RNA copies were detected by RT-qPCR using forward primer: 5’-GGCACTGACAATTCCGTGGT-3’, reverse primer: 5’-AGGGACGTAGCAGAAGGACG-3’. Then, HEK293T-ACE2 cells were seeded into 48-well plates and infected with pseudotyped viruses at equal RNA copies supplemented with polybrene (4 mg/mL). After incubation for 12 h, the pseudovirus-containing supernatant was removed and replaced with fresh DMEM containing 10 % FBS. After 72 h post-infection, the HEK293T-ACE2 cells were lysed with 50 μL passive lysis buffer (Promega, Madison, WI, USA) to measure firefly luciferase activity using a luciferase reporter assay system (Promega) according to the manufacturer’s protocol. Viral infection rate was calculated by analyzing the mean firefly luciferase activity as to control group (set as 100 %).

***Purification of SARS-CoV-2 S pseudovirus particles***

Collected the SARS-CoV-2 S pseudovirus particles containing supernatant into four 50 mL conical tubes and centrifuged for 5 minutes at 1000 rpm. Filtered the virus-containing supernatant through the 0.45 um filter flask and divided the filtered supernatant into centrifuge tubes. To the bottom of each centrifuge tube, added 4 mL of 20 % sucrose solution. Centrifuged in a Beckman SW-28 rotor for 2 hours at 25,000 rpm. Then discarded the supernatant clearly and resuspended the pellet by RIPA buffer. S protein distribution on pseudovirus particles was detected by western blot, HIV-1 p24 antigen as loading control (p24 antibody, 1:1000; Sino Biological, China).

***Lipid raft fractionations assay***

Isolation of lipid raft fractionations was performed with Lipid Raft Isolation Kit (Invent Biotechnologies, USA) in accordance with the manufacturer's instructions. In brief, HEK293T cells transfected with Flag-S were cultured on 10 cm culture dish for 48 h. Cells suspension were incubated in 500 µL buffer A on ice for 5 min and transfered the cell suspension to the filter cartridge. Capped the filter cartridge and centrifuged at 16,000 g for 30 s. Resuspended the pellet by vigorously vortexing for 10 s and centrifuged at 1,000 g for 5 min. Transferred all supernatant to a fresh 1.5 mL microfuge tube and centrifuged for 30 min at 16,000 g. The pellet is the total membrane fraction and carefully transferred all supernatant (cytosolic fraction). Resuspended the pellet in 500 µL cold buffer B and immediately incubated the tube on ice for 30 min. Centrifuged the tube at 16,000 g for 10 min and collected all supernatant. Added 0.5 mL buffer C to the tube, mixed well by vortexing and incubated the tube on ice for 2 min. Centrifuged at 10,000 g for 10 min and the lipid raft was floating on top of the tube. Carefully removed aqueous phase slowly and isolated lipid rafts were resuspended in 100 µl RIPA buffer. The cytoplasm, total membrane and lipid raft distribution were further measured by western blot, taking GAPDH as cytoplasm protein control (GAPDH antibody, 1:5000; Proteintech, USA) and Flotillin-1 as membrane and lipid raft protein control (Flotillin-1 antibody, 1:500; Santa Cruz Biotechnology, USA).

### *Western blot, Native polyacrylamide gel electrophoresis (PAGE) and Co-IP*

Western blot and Co-IP assays were performed as described previously^2^. Intensities of protein bands were determined by densitometric analysis with Image-Pro Plus software. The SARS-CoV-2 S trimerization assay was performed by native polyacrylamide gel electrophoresis as described previously^3^.

**reference**

1. Lu, Y. *et al.* Palmitoylation of NOD1 and NOD2 is required for bacterial sensing. *Science* **366**, 460-467 (2019).

2. Wang X, *et al.* ZDHHC1-mediated IFITM3 palmitoylation to inhibit Japanese encephalitis virus replication. *PLoS Pathog* **16**, e1009035 (2020).

3. Chen W, *et al.*Contribution of Ser386 and Ser396 to activation of interferon regulatory factor 3. *J Mol Biol* **379**, 251-260 (2008).

**Figure S1. Palmitoylation of SARS-CoV-2 S is necessary for cell-cell fusion and virus infection.** (A) Flag-NOD2 was transfected into HEK293T cells for 48 h and its palmitoylation was detected by ABE assay in the presence of HAM. (B) VSV-G or SARS-CoV-2 S plasmid with lentivirus packing plasmids were cotransfected into HEK293T cells for 48 h, the pseudoviruses were collected to infect HEK293T-ACE2 cells for 72 h, the GFP fluorescence and firefly luciferase activity were detected to analyse viral entry. The scale bar indicates 50 µm. (C) S-WT and S-∆C-Palm were overexpressed in HEK293T for 48 h, the cytoplasm, membrane and lipid raft distribution of S protein were detected by western blot. (D) S/GFP and S-∆C-Palm/GFP coexpressed HEK293T cells were cocultured with Dil-labelled HEK293T-ACE2 cells. Cell-cell fusion was measured with flow cytometry (*n*=3) and visualized by fluorescent imaging at the indicated time. The scale bar indicates 50 µm. One-way ANOVA, **P*<0.05; ***P*<0.01. (E) HEK293T cells were treated with 2-BP at indicated dose for 48 h, and then cell viability was measured by CCK-8 assay (*n*=3). Unpaired t-test, **P*<0.05; ***P*<0.01.
